# Supplementary material for: Contribution of narrative therapy in reduction of anxiety, depression and PTSD among survivors of the genocide against the Tutsi in 1994 in Rwanda
Source: PLOS Ment Health. 2025 Apr 7;2(4):e0000287. doi: 10.1371/journal.pmen.0000287 (PMC12798430; doi:10.1371/journal.pmen.0000287)
Supplement: Attachment — Submitted filename: Response to Reviewer .docx [file pmen.0000287.s002.docx]

**A point-by-point response to reviewers**

We sincerely appreciate the editor and reviewers for their time and effort that you have dedicated to providing insightful comments on our manuscript. We have diligently addressed all the raised issues, and we believe that the final version of this manuscript is much improved due to their valuable feedback.

**Note:** This table corresponds to the manuscript with track changes. It means that page numbers correspond to the manuscript with track changes.

| **Reviewer 1 Comments** | **Page and line on which addressed** | **How comments were addressed** |
| --- | --- | --- |
| Reviewer #1: Source 8: The publication year should be added.  Source 15: This source should also be removed as it is more than 10 years old.  Source 10: This source should be removed due to its outdated nature. | **Whole manuscript** | **Thank you for the constructive feedback.** We have thoroughly revised the manuscript, including the introduction, methods, results, discussion, and references. We used the latest version of the Mendeley referencing tool to resolve all issues related to references, including removing outdated sources and adding the publication year where missing. |
| Addressing these concerns represents a substantial amount of work. Given the extent of the issues identified, I considered issuing a reject decision. However, I think it is possible to conduct an extensive revision of your manuscript (should you choose to), so I am issuing a major revision decision instead. | **Whole manuscript** | **We deeply appreciate the opportunity to revise the manuscript.**  We have extensively revised the entire manuscript in line with your suggestions to improve clarity and coherence. |
| Clearly state the objectives, methods, results and conclusion and suggestion in abstract | **Page2, line 2** | **Thank you for your suggestion.** We have substantially revised the abstract to clearly outline the objectives, methods, results, and conclusions as follows:  **Abstract**  **Background**: The 1994 genocide against the Tutsi in Rwanda caused profound psychological trauma among survivors, with long-lasting effects such as anxiety, depression, and post-traumatic stress disorder (PTSD). Narrative therapy, known for its ability to help individuals reconstruct their personal stories, is emerging as a promising intervention, though its effectiveness within Rwandan context has not been fully explored.  **Objectives**: This study aimed to assess the impact of narrative therapy on reducing symptoms of anxiety, depression, and PTSD in genocide survivors. It focused on how narrative therapy helps survivors reconstruct their narratives, enhance resilience, and foster emotional well-being.  **Method**: Eleven qualitative interviews were conducted with survivors of the 1994 Ggenocide against the Tutsi in Rwanda who participated in narrative therapy. Through purposive sampling, participants were selected for in-depth, semi-structured interviews. The interviews were transcribed and analysed using thematic analysis to identify key themes and patterns.  **Results**: Thematic analysis revealed several key themes: survivors reported positive transformation in their narratives, which helped them reframe traumatic experiences and develop resilience. Participants strongly testified reduction in symptoms of anxiety, depression, and PTSD after engaging in narrative therapy. The communal aspects of therapy, such as group support, were also instrumental in fostering emotional recovery and a renewed sense of belonging.  **Conclusion:** Narrative therapy appears to provide an effective framework for addressing psychological distress in genocide survivors by offering a structured process for narrative reconstruction and community healing. Further research is needed to confirm these findings and explore long-term outcomes. Culturally relevant mental health interventions, supported by sustainable funding, are essential to improving survivors' well-being and fostering resilience.  ***Keywords:*** *Anxiety, Depression, Genocide against Tutsi, Narrative therapy, PTSD, Reduction* |
| Introduction is very week only focusing on the nature of narrative therapy while citing a few studies from the contexts other than the current study’s context. It is recommended to first **provide the background** **of the Tutsi genocide while contextualizing the terms in local including genocide, trauma/violence, mental health, mental disorders so on and so forth.** |  | **Thank you for the insightful comment.**  We have revised the introduction to provide a more comprehensive background on the genocide, its psychological impacts, and the prevalence of trauma-related disorders in Rwanda. We have contextualized terms like trauma and mental health within the local context and expanded the literature review to provide a more robust foundation for the study. **We rigorously have revised the introduction as follows:**  **Introduction**  The 1994 genocide against the Tutsi in Rwanda was one of the most horrific and devastating events of the 20th century, with over 800,000 people killed in just 100 days[1]. Many genocide survivors continue to experience severe mental health disorders, particularly post-traumatic stress disorder (PTSD), depression, and anxiety [2,3]. The impact of these mental health conditions is both profound and persistent, affecting not only those who directly witnessed or survived the violence but also subsequent generations [4–7]. Addressing these psychological consequences has become a critical priority in Rwanda's mental health care system, particularly due to the prevalence of trauma-related disorders among the genocide survivors.  Research consistently highlights PTSD, depression, and anxiety as the most common mental health disorders affecting genocide survivors of the 1994 genocide against the Tutsi. In a national study, scholars reported that the most prevalent mental health conditions among Rwandans genocide survivors are major depressive episodes (35%), PTSD (27.9%), and panic disorder (26.8%) in Rwanda [8]. Similarly, recent study found that 37% of female survivors continued to suffer from PTSD [3]. These findings underscore the pressing need for therapeutic interventions to address the enduring impact of these mental health conditions.  Despite the high prevalence of these disorders, access to mental health care remains limited in Rwanda, particularly in rural areas where many survivors reside [9]. In response, narrative therapy has emerged as a promising intervention. Developed in the 1980s, narrative therapy is based on the idea that people make sense of their lives through the stories they tell about themselves [10]. This therapeutic approach encourages individuals to externalize their problems—viewing them as separate from themselves—and then to re-author their personal narratives in ways that emphasize resilience, empowerment, and healing [10],. Narrative therapy’s focus on "meaning-making" is especially relevant in trauma recovery, where individuals often struggle to process their traumatic experiences and construct coherent narratives about their suffering [11].  In Rwanda, the cultural practice of oral storytelling plays a central role in both personal and collective identity formation. Narrative therapy aligns with these traditions by providing a structured way for survivors to reconstruct their life stories, emphasizing survival and strength [12]. By engaging with narrative therapy, survivors have the opportunity to reframe their experiences in ways that promote healing on both individual and communal levels, making it an especially suitable intervention in the Rwandan context.  Although research on narrative therapy in Rwanda is limited, existing studies highlight its potential. Authors have recently found that narrative therapy significantly improve resilience and reduce anxiety and ADHD in randomized controlled trials conducted among Rwandan orphan children [13,14]. However, much of the research on narrative therapy’s effectiveness has been conducted outside of Rwanda. For instance, a meta-analysis by Lely et al.[15] found medium to large effect sizes for narrative therapy in treating PTSD and depression, enabling participants to reframe their traumatic experiences and regain a sense of agency. Scholars similarly reported its effectiveness in addressing various mental health issues in children, such as attachment disorders and family violence [16,17]. Studies involving war refugees [18] and other traumatized populations [19,20] also found that narrative therapy significantly reduced PTSD symptoms and helped trauma survivors reconstruct their identities.  Despite the demonstrated potential of narrative therapy in various populations, there is a significant research gap regarding its specific impact on adult Rwandan genocide survivors. Most studies [13,14] have focused on children and orphans, leaving limited evidence on how narrative therapy affects adults suffering from PTSD, depression, and anxiety. Moreover, while the meta-analysis by Lely et al.[15] supports narrative therapy’s effectiveness in treating trauma, there is a lack of context-specific studies that consider Rwanda’s unique cultural and historical background. Addressing this gap requires research that adapts narrative therapy to the specific needs of adult survivors, with a focus on long-term trauma effects and the potential for collective healing.  Given the high prevalence of PTSD, depression, and anxiety among genocide survivors and the strong cultural resonance of storytelling in Rwanda, this study seeks to fill this critical gap in the literature by examining the impact of narrative therapy on these three prevalent mental health conditions. By exploring how narrative therapy can facilitate individual and communal healing, this research aims to contribute to the development of culturally relevant mental health interventions in Rwanda, with important implications for mental health policies and practices in post-genocide settings. |
| The authors should cite the relevant literature from other parts of the world. |  | **We appreciate the suggestion.**  We have added relevant global literature on the effectiveness of narrative therapy in different contexts, including studies on trauma, PTSD, and mental health interventions in various populations, such as war refugees and trauma survivors in other regions as follows:  Although research on narrative therapy in Rwanda is limited, existing studies highlight its potential. Authors have recently found that narrative therapy significantly improve resilience and reduce anxiety and ADHD in randomized controlled trials conducted among Rwandan orphan children [13,14]. However, much of the research on narrative therapy’s effectiveness has been conducted outside of Rwanda. For instance, a meta-analysis by Lely et al.[15] found medium to large effect sizes for narrative therapy in treating PTSD and depression, enabling participants to reframe their traumatic experiences and regain a sense of agency. Scholars similarly reported its effectiveness in addressing various mental health issues in children, such as attachment disorders and family violence [16,17]. Studies involving war refugees [18] and other traumatized populations [19,20] also found that narrative therapy significantly reduced PTSD symptoms and helped trauma survivors reconstruct their identities. |
| The term broad objective should be used instead of general objective |  | **Thank so much for the comment, it was corrected as follows.**  Given the high prevalence of PTSD, depression, and anxiety among genocide survivors and the strong cultural resonance of storytelling in Rwanda, this study seeks to fill this critical gap in the literature by examining the impact of narrative therapy on these three prevalent mental health conditions. By exploring how narrative therapy can facilitate individual and communal healing, this research aims to contribute to the development of culturally relevant mental health interventions in Rwanda, with important implications for mental health policies and practices in post-genocide settings. |
| The nature of psycho pathological symptoms developed should also be provided by citing the literature from Rwanda resulting from trauma and violence  Why authors have focused only on the anxiety, depression and PTSD only? |  | We addressed this by providing a detailed explanation of the prevalence of these specific mental health conditions among genocide survivors in Rwanda, supported by local and global research. These conditions were chosen due to their prominence in trauma literature and their significant impact on the population. The following was added in the manuscript:  The 1994 genocide against the Tutsi in Rwanda was one of the most horrific and devastating events of the 20th century, with over 800,000 people killed in just 100 days[1]. Many genocide survivors continue to experience severe mental health disorders, particularly post-traumatic stress disorder (PTSD), depression, and anxiety [2,3]. The impact of these mental health conditions is both profound and persistent, affecting not only those who directly witnessed or survived the violence but also subsequent generations [4–7]. Addressing these psychological consequences has become a critical priority in Rwanda's mental health care system, particularly due to the prevalence of trauma-related disorders among the genocide survivors.  Research consistently highlights PTSD, depression, and anxiety as the most common mental health disorders affecting genocide survivors of the 1994 genocide against the Tutsi. In a national study, scholars reported that the most prevalent mental health conditions among Rwandans genocide survivors are major depressive episodes (35%), PTSD (27.9%), and panic disorder (26.8%) in Rwanda [8]. Similarly, recent study found that 37% of female survivors continued to suffer from PTSD [3]. These findings underscore the pressing need for therapeutic interventions to address the enduring impact of these mental health conditions. |
| The interview schedule was "self-constructed", meaning it was developed by the authors? If so, was the interview schedule informed by the literature or just questions that the authors came up with? Please justify. |  | Yes, the interview schedule was self-constructed, informed by extensive literature on trauma, narrative therapy, and mental health in post-genocide settings. This was clarified in the revised methods section as follows:  Data were gathered using a semi-structured interview guide, which was developed by the authors and informed by relevant literature on trauma and narrative therapy. Data were gathered using a semi-structured interview guide, which was developed by the authors and informed by relevant literature on trauma and narrative therapy. The guide’s questions were designed to explore the impact of narrative therapy on mental health outcomes such as PTSD, depression, and anxiety, while allowing flexibility for participants to express their personal stories. Sample questions included: “How would you describe the problem that has affected you, and what can you compare it to?”, “Could you share your experiences participating in therapeutic sessions?”, “Can you compare your situation before and after attending therapeutic sessions?” Probing questions, such as, “can you tell me what you felt about that?” Interviews lasted between 45 to 60 minutes and were audio-recorded with participant consent to ensure accuracy in data collection. |
| The 10 themes were derived from the interviews conducted; the authors should examine whether they could be further categorized into sub-themes.  Moreover, the themes should be further framed within a framework or model that functionally speaks to adjustment of the participants.  The authors are suggested to write the themes more coherently and provide numbering to the themes by providing the verbatim responses while explaining the themes to aid the readers. |  | Thank you for the suggestion. We revisited the data and identified sub-themes for each main theme. We also provided a clearer narrative around these themes, grouping them where relevant, and presented them in a more coherent manner within a framework that reflects participants' psychological adjustment. We have also included verbatim responses from participants under each theme to offer a more vivid representation of their experiences and to support the analysis. The results section was rigorously revised as follows: **3. Results** **3.1 Sociodemographic characteristics of the participants** Table 1 summarizes the demographic characteristics of 11 participants, ranging in age from 31 to 75 years old (M=49.5, SD=14.6), with the majority being female (7/11). Most participants have a primary education (7/11), two have secondary education and two are illiterate. Five participants are widows, reflecting the impact of historical events like the genocide. All participants are jobless, suggesting economic difficulties, and there is an even split between urban (5/11) and rural (6/11) residents. These characteristics provide important context for understanding how factors such as gender, education, and marital status may influence participants' mental health and engagement in narrative therapy. **Table 1: Sociodemographic characteristics of the participants**  **3.2 Understanding the Multifaceted Experiences of survivors of the 1994 genocide against the Tutsi.** Our analysis identifies ten major themes that capture the complex and multifaceted experiences of the genocide survivors, from their initial engagement with psychological care to the therapeutic processes that helped them cope. These themes also highlight the challenges related to trauma, as well as the restorative impact of therapeutic interventions. Below, we present these themes, along with corresponding subthemes, frequencies, and verbatims to provide deeper insights into the survivors' lived experiences. **Table 2: Summary of themes and sub-themes** **Themes** \| **Sub-themes** \| **Frequency**; **1. Initiation to Psychological Care Process** \| \|; \| - Self-initiated \| 4/11; \| - External figures (Ibuka president, friend, family) \| 7/11; \| - Learned through community programs, word of mouth \| 6/11; **2. Reasons for Psychological Care** \| \|; \| - Insomnia \| 6/11; \| - Flashbacks \| 5/11; \| - Feeling disconnected from family and community \| 7/11; \| - Seeking connection with others \| 7/11; \| - Physical symptoms (e.g., headaches, fatigue) \| 6/11; **3. Naming of Problems and Descriptions** \| \|; \| - Described trauma using metaphors (e.g., hell, darkness) \| 8/11; \| - Likened trauma to death or deep emotional pain \| 7/11; \| - Difficulty finding words to describe emotional pain \| 5/11; **4. Experiences of Complex Trauma** \| \|; \| - Recurrent memories of rape or multiple sexual assault \| 5/11; \| - Continued psychological pain and suffering \| 9/11; \| - Social rejection from community, neighbours, family \| 6/11; \| -Re-traumatization during commemoration events \| 6/11; **5. Perceptions of Therapeutic Setting** \| \|; \| - Found comfort in shared group experiences \| 5/11; \| - Preferred individual therapy for deeper discussions \| 6/11; \| - Safe space for emotional expression and breakdowns \| 8/11; \| - Hesitancy to disclose sensitive issues in group therapy. \| 4/11; **6. Outcomes Following Narrative Therapy** \| \|; \| - Better management of emotions, calming techniques \| 9/11; \| - Re-established communication with family members \| 6/11; \| - Restoring a Sense of Control over life \| 7/11; **7. Restored Trust Among Neighbors** \| \|; \| - Slowly rebuilding trust in neighbours \| 6/11; \| - Overcoming fear of neighbours implicated in violence \| 5/11; \| - Developing empathy for neighbors’ situations \| 4/11; \| - Cautious trust, but hopeful for better relations \| 5/11; \| - Instances of forgiveness between neighbors \| 4/11; **8. Instilling Hope** \| \|; \| - Renewed ability to plan for the future \| 7/11; \| - Found new sense of purpose through therapy \| 5/11; \| - Expressed hope for better outcomes for children \| 4/11; \| - Overcoming Suicidal Thoughts \| 3/11; \| - Learning coping strategies for future challenges \| 7/11; **9. Sense of Family Belonging** \| \|; \| - Rebuilt connections with family members \| 5/11; \| - Found a new family within the therapy group \| 6/11; \| - Mending Relationships with Children \| 4/11; \| - Restored a sense of identity and belonging \| 5/11; **10. What Truly Transpired During Therapy** \| \|; \| - Healing through telling their stories \| 8/11; \| - Guided reflection leading to breakthroughs \| 6/11; \| - Learned practical techniques for managing emotions \| 7/11; \| - Group activities helped rebuild trust \| 5/11 **The following are the sample description of the themes (three themes), but we did the same for all themes:** **3.2.1. Initiation to Psychological care process** Participants described diverse pathways through which they accessed psychological care, including self-initiation and external influences from community members. Local leaders, friends, and community health workers often played a critical role in encouraging survivors to seek therapeutic support. In several cases, the consultation was initiated by these community figures. For example, Participant **MUH** shared, **"*The president of Ibuka was the one who called me and initiated the consultation."*** Another participant, **ESP,** mentioned **"*It was through the Croix Rouge that I was invited for follow-up sessions after they noticed we had emotional crises at a commemoration event.*"** Other participants sought psychological support after recognizing the severity of their emotional and physical distress, marked by trauma symptoms such as insomnia, flashbacks, headaches, and bodily pain. As **MUK** recounted, ***"I felt so weak and could barely move. I had constant headaches, and the memories of what happened kept coming back. That’s when I knew I needed help.*" UWI** reflected, **"*A friend who was in the same group explained my problem and encouraged me to seek help. That's when I decided to consult.*"** **3.2.2. Reasons for search of psychological care** Participants provided several reasons for seeking psychological care, primarily driven by intense emotional and physical distress that disrupted their daily lives. This distress often included symptoms of depression, anxiety, or PTSD. For many, these symptoms were exacerbated during genocide commemoration periods, where memories of past trauma resurfaced. **MUH** described how her experience: **"*I had trauma-related symptoms like insomnia, confusion, and anger, especially during the commemoration period. I preferred staying at memorial sites because I felt relieved, but when I went home, I heard voices and couldn’t sleep."*** Similarly, **VTT** shared, ***"I experienced outbursts of anger and insomnia, and when I was asked too many questions, I would become silent and absent-minded."*** For some, physical symptoms prompted them to seek care. Participant **NYD** explained, ***"I felt hot, had severe headaches, and my neck felt like hot liquid was flowing down it. Those physical symptoms made me realize something was wrong."*** Others, like **ESP**, expressed fatigue and physical weakness, stating, ***"I was always tired and didn’t have the energy to do anything. That’s when I realized I needed help."*** Many participants noted that social withdrawal pushed them to seek therapy. MKT shared, ***"I had isolated myself from my children. I was angry all the time and I didn’t want to interact with anyone."*** MUH similarly noted, ***"I stopped talking to my neighbours and preferred staying alone at home."*** Participants also sought emotional relief and support, hoping to connect with others to alleviate their burdens. **MKT** said, ***"I wanted to feel like I wasn’t alone. That’s why I went to the psychologist. I needed to talk to someone who could help me understand what I was going through."*** 2.2.9 **Sense of family belonging** Therapy facilitated the restoration of family connections and helped participants feel a renewed sense of belonging, both within their biological families and their therapeutic group. **MUH** reflected, ***"Before, I distanced myself from my family because I didn’t want them to see my pain. But therapy has helped me reconnect with them."*** For some, group therapy became a substitute for the family they had lost. ESP explained, ***"In the therapy group, we became like a family. We supported each other, and that made all the difference."*** **DEN** shared a similar sentiment: ***"I never thought I’d find a family again, but this group has given me a sense of belonging."*** Participants also discussed how therapy helped them repair relationships with their children. MKT shared, ***"Before therapy, I couldn’t even talk to my children. Now we play together and talk about what’s going on in our lives."*** Additionally, many participants shared how this renewed sense of belonging helped restore their sense of identity. VTT noted, ***"I felt like I was nobody after everything that happened, but being part of this group has given me a new sense of who I am."*** |
| The biggest drawback of the paper is that the findings have not been discussed therefore, it is recommended to adequately discuss the findings while citing relevant literature. |  | **Thank you so much for the constructive comment.**  The discussion has been thoroughly revised to include more in-depth analysis, drawing on relevant literature. We have framed our findings within existing research on trauma recovery and narrative therapy, both in Rwanda and globally. It was revised as follows:  **4. Top of Form**  **Discussion**  This study explored the experiences of survivors of the 1994 genocide against the Tutsi in Rwanda, focusing on their initiation into psychological care and the outcomes of narrative therapy. The findings are particularly significant given Rwanda's historical context and the ongoing mental health challenges that genocide survivors face. Over 25 years after the genocide, many survivors continue to suffer from PTSD, depression, and anxiety [2,8]. This study contributes to the growing body of literature by providing an in-depth understanding of how narrative therapy, a culturally relevant intervention, impacts the mental health of adult genocide survivors.  The initiation of psychological care among survivors was largely influenced by community figures, which underscores the vital role of social networks in post-genocide Rwanda. Survivors often rely on community leaders or local organizations to facilitate their access to mental health services, particularly in rural areas where formal mental health infrastructure remains limited [9]. This aligns with findings by Rieder & Elbert [4] and extends the understanding of how physical symptoms, such as headaches, fatigue, and insomnia, often prompt survivors to seek psychological help. This finding is consistent with Van der Kolk’s work on trauma, which emphasizes the manifestation of trauma through both physical and emotional symptoms[26]. In Rwanda's context, where physical health often takes precedence, addressing these somatic symptoms in conjunction with psychological ones becomes imperative for holistic trauma care.  Survivors’ reasons for seeking psychological help were primarily rooted in severe emotional distress, which was often exacerbated during annual genocide commemoration periods. The commemoration events serve as a trigger for the reactivation of traumatic memories, a pattern documented in other studies [28,29]. Our findings highlight the need for targeted mental health interventions during these emotionally charged times. Participants frequently described how physical manifestations of trauma, such as body pain and fatigue, were intertwined with their psychological distress, contributing to a cycle of suffering. This aspect of trauma, where physical and emotional symptoms are inseparable, has been extensively discussed in trauma literature, including Van der Kolk’s work on the body’s role in storing traumatic memories [26].  When it came to describing their trauma, many participants struggled to find the words to articulate their experiences. This difficulty in verbalizing trauma is a well-documented phenomenon in trauma studies [30]. Our study builds on this by showing how Rwandan survivors use culturally specific metaphors to describe their pain, such as likening their suffering to "dying without dying." This use of metaphor is particularly relevant in Rwanda, where oral storytelling is central to cultural identity [12]. The integration of narrative therapy, which relies on storytelling, aligns with these cultural practices, making it a particularly suitable intervention for Rwandan survivors. Future research should explore how the use of metaphors in therapeutic settings evolves over time and whether it aids in the processing of trauma.  Participants in this study also reported ongoing psychological distress related to their experiences of the genocide, particularly those who had endured extreme acts of violence, such as rape, torture, and the loss of loved ones. This is consistent with literature on complex trauma, which emphasizes the long-term psychological consequences of such experiences [31,32]. The emotional numbness, hypervigilance, and detachment described by participants echo the symptoms of PTSD, as documented in both international and Rwandan studies [33,34]. What our study adds is a deeper understanding of how these long-term trauma effects hinder survivors' reintegration into society and their ability to rebuild relationships. The isolation felt by survivors is not only social but emotional, emphasizing the importance of sustained psychological interventions that address deep-seated emotional wounds.  The mixed perceptions of the therapeutic setting, with some participants finding comfort in group therapy and others preferring individual sessions, highlight the need for flexible therapy models. While group therapy provides a sense of solidarity and shared experience, which is particularly valuable in post-conflict settings [35,36], some survivors feel hesitant to share sensitive issues in such settings. These findings underscore the importance of creating adaptable therapeutic environments that offer both group and individual therapy sessions, depending on the survivor's needs. Future research could investigate how survivors’ preferences for therapeutic formats evolve over time and whether their comfort with certain formats increases as their healing progresses.  Narrative therapy was shown to have a positive impact on survivors, particularly in terms of emotional regulation and reconnection with family members. This aligns with research on the effectiveness of narrative therapy in treating trauma, as it enables survivors to reframe their experiences and regain a sense of control over their narratives[10,15,37,38]. The storytelling aspect of narrative therapy resonates deeply with Rwandan cultural traditions, where oral storytelling is a key part of identity formation [12]. This study adds to the existing literature by detailing the therapeutic techniques that survivors found most effective—such as storytelling, guided reflections, and grounding exercises—showing the importance of these techniques in facilitating emotional breakthroughs and long-term healing.  One of the most profound findings of this study is the restoration of trust between survivors and their neighbors, including in cases where neighbors were involved in the genocide. This finding emphasizes the role of therapy in facilitating reconciliation, a topic explored in previous research [39]. Participants spoke of forgiveness and rebuilding relationships, showing how therapy can play a crucial role in community-level healing, not just individual recovery. Further research should investigate how these reconciliation efforts are sustained over time and how broader societal factors, such as government reconciliation programs, contribute to or hinder this process.  A renewed sense of hope was another key outcome of narrative therapy. This finding supports previous research on the role of hope in trauma recovery [40,41]. Survivors in this study felt more in control of their emotions and futures, with therapy helping them develop practical coping strategies. These outcomes align with studies that emphasize the role of narrative therapy in fostering resilience and empowerment [11,16]. The long-term focus of this study provides new insights into how survivors maintain hope and resilience in the face of ongoing challenges, highlighting the importance of continued mental health support in post-genocide contexts.  In addition to emotional healing, therapy helped survivors restore a sense of family belonging, whether with their biological families or through the therapeutic group. This finding aligns with previous research emphasizing the role of family connections in trauma recovery [42,43]. For many survivors, who lost family members during the genocide, the therapeutic group became a substitute family, providing much-needed emotional support. This study adds to the literature by showing how these therapeutic group relationships can provide long-term emotional support, creating new "families" for survivors who have lost their original ones.  Finally, participants reflected on the specific therapeutic techniques that were most beneficial to their healing, including storytelling, guided reflections, and emotional breakthroughs. These moments of emotional release were key in helping survivors process their trauma and move forward, which supports previous research on the effectiveness of narrative therapy in addressing trauma-related mental health issues [15]. Future research should explore how these techniques can be refined to better address the specific needs of Rwandan survivors, ensuring that therapeutic interventions remain culturally relevant and effective.  **Strengths and Limitations**  This study has several strengths. It provides a detailed, nuanced understanding of the long-term effects of trauma and the role of narrative therapy in the healing process for genocide survivors. The study also emphasizes the importance of culturally sensitive interventions, offering insights into how narrative therapy aligns with Rwanda's oral storytelling traditions. However, the study has limitations. The reliance on self-reported data may introduce recall bias, as participants might have difficulty accurately remembering or might selectively report their experiences. Additionally, the cross-sectional design limits the ability to assess long-term changes in mental health outcomes. Furthermore, the study focuses on adult survivors, leaving a gap in understanding how younger generations affected by the genocide may respond to similar interventions. Finally, as qualitative study it focused on a specific geographic region, which may limit the generalizability of the findings to other survivor populations across Rwanda. |
| It is also recommended to provide separate sections for limitations, implication and/or suggestions which are the essence of this kind of study from a specific context. This has made the paper very week. |  | **Thank you so much for the insightful comment.**  We have added a separate section for strengths, limitations, and implications. This section discusses the study's contributions, its limitations (such as the cross-sectional design and reliance on self-reported data), and the implications for future research and practice, especially in developing culturally relevant mental health interventions for genocide survivors. The following was added in the manuscript.  **Strengths and Limitations**  This study has several strengths. It provides a detailed, nuanced understanding of the long-term effects of trauma and the role of narrative therapy in the healing process for genocide survivors. The study also emphasizes the importance of culturally sensitive interventions, offering insights into how narrative therapy aligns with Rwanda's oral storytelling traditions. However, the study has limitations. The reliance on self-reported data may introduce recall bias, as participants might have difficulty accurately remembering or might selectively report their experiences. Additionally, the cross-sectional design limits the ability to assess long-term changes in mental health outcomes. Furthermore, the study focuses on adult survivors, leaving a gap in understanding how younger generations affected by the genocide may respond to similar interventions. Finally, as qualitative study it focused on a specific geographic region, which may limit the generalizability of the findings to other survivor populations across Rwanda.    **The implication of this study was added at the end of the conclusion as follows:**  **Conclusion**  The analysis of the participant interviews highlights the complex emotional and psychological journeys of survivors of the 1994 genocide against the Tutsi, underscoring the critical role that psychological care, particularly narrative therapy, in the healing process. Providing safe spaces for survivors to share their experiences, process their emotions, and reconnect with families and communities has proven effective in alleviating symptoms of anxiety, depression, and PTSD. Therapeutic techniques such as storytelling, guided reflection, and group support facilitated emotional breakthroughs, restore family bonds, and offered survivors renewed hope. The communal aspects of therapy were particularly powerful, as many participants reported a renewed sense of belonging and trust within their communities, highlighting the importance of incorporating social and community-based healing approaches in trauma recovery.  **Expanding psychological care programs that integrate narrative therapy and community-based healing across Rwanda is essential to increase survivor access. Mental health professionals should be trained in culturally appropriate narrative therapy and group interventions to enhance therapeutic effectiveness. Policymakers must prioritize sustainable, long-term funding for trauma recovery programs to ensure continuity of care. Additionally, further comparative studies with larger sample sizes are recommended to comprehensively assess the therapeutic efficacy of narrative therapy and explore its integration with other approaches for greater impact on emotional regulation, family reconciliation, and community reintegration.** |
