# Supplementary material for: Contribution of narrative therapy in reduction of anxiety, depression and PTSD among survivors of the genocide against the Tutsi in 1994 in Rwanda
Source: PLOS Ment Health. 2025 Apr 7;2(4):e0000287. doi: 10.1371/journal.pmen.0000287 (PMC12798430; doi:10.1371/journal.pmen.0000287)
Supplement: Attachment — Submitted filename: Response to reviewers.docx [file pmen.0000287.s004.docx]

**A point-by-point response to reviewers**

We sincerely appreciate the editor for his/her time and effort dedicated to providing insightful comments on our manuscript. We have diligently addressed all the raised issues, and we believe that the final version of this manuscript is much improved due to his/her valuable feedback.

**Note:** This table corresponds to the manuscript with track changes. It means that page numbers correspond to the manuscript with track changes.

| **Editer 1 Comment** | **Page and line on which addressed** | **How comments were addressed** |
| --- | --- | --- |
| I have one suggestion to improve the manuscript. In the section on data collection and procedure, the authors state that " Data were gathered using a semi-structured interview guide, which was developed by the authors and informed by relevant literature on trauma and narrative therapy". Can the authors please cite the articles that they referred to when they developed the interview guide. | **Page 6, Line 149** | Thank you so much for the insightful comment. It has been addressed as follows:  *“Data were gathered using a semi-structured interview guide, which was developed by the authors and informed by relevant literature on trauma and narrative therapy [10, 15, 25, 26]. The guide’s questions were designed to explore the impact of narrative therapy on mental health outcomes such as PTSD, depression, and anxiety, while allowing flexibility for participants to express their personal stories.”*  *Below are the specific references that informed the development of the interview guide:*   10. ***Groleau D, Young A, Kirmayer LJ.*** *The McGill Illness Narrative Interview (MINI): An Interview Schedule to Elicit Meanings and Modes of Reasoning Related to Illness Experience.* Transcultural Psychiatry. *2006;43: 671–691. doi:10.1177/1363461506070796*     a. *This resource provided the foundational structure for eliciting detailed personal narratives and exploring the meanings participants attach to their experiences of illness and trauma.*   ***15. Lely JCG, Smid GE, Jongedijk RA, Knipscheer JW, Kleber RJ.*** *The effectiveness of narrative exposure therapy: A review, meta-analysis, and meta-regression analysis.* European Journal of Psychotraumatology. *2019;10. doi:10.1080/20008198.2018.1550344*   a. *This article contributed insights into the therapeutic effects of narrative exposure therapy, shaping questions that address both past traumatic experiences and the impact of therapeutic interventions.*   ***25. White M, Epston D.*** *Narrative means to therapeutic ends. New York: WW Norton & Company; 1990.*   b. *This foundational work on narrative therapy emphasized the importance of externalizing problems and re-authoring personal stories, which informed the thematic focus of the interview guide.*  26. ***Neuner F, Elbert T, Schauer M.*** *Narrative exposure therapy for PTSD. In: Casebook to the APA Clinical Practice Guideline for the Treatment of PTSD. Washington: American Psychological Association; 2020. pp. 187–205. doi:10.1037/0000196-009*     a. *This reference guided the inclusion of questions aimed at uncovering specific traumatic memories and participants’ reflections on their therapeutic journeys.*   *By integrating these key references, the interview guide was designed to ensure a robust and methodologically sound approach to exploring participants' experiences with trauma and their engagement in therapeutic processes.* |
